# Testing for Lynch Syndrome in Endometrial Carcinoma: From Universal to Age-Selective *MLH1* Methylation Analysis

**DOI:** 10.3390/cancers14051348

**Published:** 2022-03-06

**Authors:** Annukka Pasanen, Mikko Loukovaara, Elina Kaikkonen, Alisa Olkinuora, Kirsi Pylvänäinen, Pia Alhopuro, Päivi Peltomäki, Jukka-Pekka Mecklin, Ralf Bützow

**Affiliations:** 1 Department of Pathology, Helsinki University Hospital, University of Helsinki, 00290 Helsinki, Finland; ralf.butzow@hus.fi; 2 Department of Obstetrics and Gynecology, Helsinki University Hospital, University of Helsinki, 00290 Helsinki, Finland; mikko.loukovaara@hus.fi; 3 Laboratory of Genetics, HUS Diagnostic Center, Helsinki University Hospital, University of Helsinki, 00209 Helsinki, Finland; elina.kaikkonen@hus.fi (E.K.); pia.alhopuro@hus.fi (P.A.); 4 Department of Medical and Clinical Genetics, University of Helsinki, 00014 Helsinki, Finland; alisa.olkinuora@helsinki.fi (A.O.); paivi.peltomaki@helsinki.fi (P.P.); 5 Department of Education and Science, Central Finland Health Care District, 40620 Jyväskylä, Finland; kirsi.pylvanainen@ksshp.fi; 6 Department of Surgery, Central Finland Health Care District; 40620 Jyväskylä, Finland; jukka-pekka.mecklin@ksshp.fi; 7 Department of Sport and Health Sciences, Jyväskylä University, 40014 Jyväskylä, Finland; 8 Applied Tumor Genomics Research Program, University of Helsinki, 00290 Helsinki, Finland

**Keywords:** endometrial carcinoma, Lynch syndrome screening, MLH1 immunohistochemistry, *MLH1* methylation analysis

## Abstract

**Simple Summary:**

International guidelines recommend universal screening of endometrial carcinoma patients for Lynch syndrome, a hereditary cancer predisposition syndrome. Screening is based on mismatch repair protein immunohistochemistry and reflex *MLH1* methylation analysis to exclude the likely sporadic cases of MMR deficiency. As sporadic MLH1 protein loss is common in endometrial carcinoma, the ability to target methylation testing would save efforts and costs. We discovered that limiting methylation testing to patients under 65 years would have significantly reduced the testing effort while maintaining a low false negative rate for *MLH1*-LS detection (0% and 3% in our clinic and registry-based cohorts, respectively).

**Abstract:**

International guidelines recommend universal screening of endometrial carcinoma (EC) patients for Lynch syndrome (LS). This screening is generally based on mismatch repair (MMR) protein immunohistochemistry followed by *MLH1* methylation analysis of MLH1-negative cases to exclude the likely sporadic cases from germline testing. As LS-associated EC is uncommon in the elderly, age-selective methylation testing could improve cost-efficiency. We performed MMR immunohistochemistry on 821 unselected ECs (clinic-based cohort) followed by a *MLH1* promoter methylation test of all MLH1/PMS2-negative tumors. Non-methylated MLH1-deficient cases underwent NGS and MLPA-based germline analyses to identify *MLH1* mutation carriers. A reduction in the test burden and corresponding false negative rates for LS screening were investigated for various age cut-offs. In addition, the age distribution of 132 *MLH1* mutation carriers diagnosed with EC (registry-based cohort) was examined. A germline *MLH1* mutation was found in 2/14 patients with non-methylated MLH1-deficient EC. When compared to a universal methylation analysis, selective testing with a cut-off age of 65 years, would have reduced the testing effort by 70.7% with a false negative rate for LS detection of 0% and 3% in the clinic and registry-based cohorts, respectively. The use of age-selective methylation analysis is a feasible way of reducing the costs and laboratory burden in LS screening for EC patients.

## 1. Introduction

Approximately 3% of endometrial carcinoma (EC) cases are associated with Lynch syndrome (LS), a cancer predisposition syndrome previously referred to as hereditary non-polyposis colorectal cancer (HNPCC) [1]. Individuals with LS have inherited a dysfunctional germline allele of a DNA mismatch repair (MMR) gene (*MLH1, PMS2, MSH2*, or *MSH6*). A secondary somatic inactivation of the remaining wild type allele leads to disruption of the MMR system and microsatellite instability (MSI). Microsatellites are DNA regions containing repeated units of 1–6 nucleotides, which are particularly prone to replication errors and an accumulation of mutations in the absence of a functional MMR system. MSI promotes carcinogenesis in various organs including the colorectum, uterus, ovary, small intestine, stomach and upper uroepithelial tract [2]. Cumulative incidence of EC in female carriers of LS is comparable to that of colorectal carcinoma, i.e., up to 50% varying by specific mutations [3]. EC represents the sentinel cancer in 50% of female LS patients with multiple malignancies [4]. In addition to possible hereditary aspects, the hypermutated MSI phenotype has prognostic implications in EC as established by The Cancer Genome Atlas (TCGA) in 2013 [5]. Accordingly, current international guidelines recommend universal tumor-based MMR analysis for all new EC cases [6,7].

MSI/LS screening is generally based on immunohistochemistry, which detects the loss of MMR protein/proteins in the tumor tissue. Molecular MSI analysis is also used, but as a disadvantage, this technique does not provide information on the specific MMR gene to be tested. Further, its sensitivity may be lower given the relatively high frequency of MSH6 loss in EC and the typically lower level of MSI caused by the inactivation of this particular gene [8,9]. As only 10% of all MMR deficient ECs are associated with germline mutation, immunohistochemistry alone is not enough to diagnose LS [1]. The majority of MMRd ECs are associated with loss of MLH1, which is generally a sporadic alteration [10,11]. *MLH1* promoter hypermethylation, on the other hand, is uncommon in LS-associated tumors. It has been reported in approximately 5% of LS colorectal carcinomas but only in isolated cases of EC, all of which were associated with constitutional epimutation (germline *MLH1* hypermethylation), a rare etiology of LS with non-Mendelian inheritance [12,13,14,15]. Therefore, MLH1 immunohistochemistry and reflex methylation analysis can be used as surrogate markers to confirm the likely sporadic origin of the tumoral MLH1 deficiency. Recently updated ESGO-ESTRO-ESP and NCCN guidelines recommend genetic counseling and subsequent germline mutation analysis for patients with MLH1/PMS2 deficient, non-*MLH1* promoter hypermethylated tumors, and to those with a loss of MMR proteins other than MLH1, in order to identify LS [7,16].

As MLH1 deficiency is detected in over 20% of unselected EC samples, reflex methylation testing generates substantial costs and burdens on laboratories [17,18]. Because LS cancer patients are typically younger, the question of whether LS screening and specifically *MLH1* methylation analysis could be limited to patients under a certain age limit arises. The objective of our study was to examine the performance of a *MLH1*-LS screening method based on immunohistochemistry and age selective *MLH1* methylation analysis. The age of cancer occurrence in Finnish *MLH1* mutation carriers was explored in an unselected clinic-based EC cohort and in a LS registry-based cohort. 

## 2. Materials and Methods

A tissue microarray (TMA) was constructed on 842 primary tumor samples from patients who underwent primary surgical treatment for stage I-IV endometrial cancer at the Department of Obstetrics and Gynecology, Helsinki University Hospital between 2007 and 2012 [19]. Patients with MLH1-negative EC and successful *MLH1* methylation analysis formed our clinic-based cohort. The registry-based cohort consisted of 132 *MLH1* mutation carriers diagnosed with EC who were identified through the Lynch Syndrome Registry of Finland (LSRFi, accessed on 1 September 2021). The LSRFi is a nation-wide research registry (est. 1982) operating in Jyväskylä and Helsinki, Finland, that organizes surveillance and cancer prevention for LS families. 

The following monoclonal antibodies were used for chromogenic immunohistochemistry on multicore TMA slides: MLH1 (ES05, Dako), MSH2 (G219–1129, BD Biosciences), MSH6 (EPR3945, Abcam), PMS2 (EPR3947, Epitomics). The slides were scored by a pathologist blinded to the clinical data. A second investigator (R.B.) examined equivocal cases and a consensus was reached. A mismatch repair protein status was considered deficient (MMR-D) when we observed a complete loss of nuclear expression in the carcinoma cells of 1 or more MMR proteins (MLH1, MSH2, MSH6, PMS2) detected by immunohistochemistry. MMR proteins form heterodimer complexes (MLH1/PMS2 and MSH2/MSH6) and only MLH1 and MSH2 are stable without their dimer partners. Hence, tumors showing the loss of both MLH1 and PMS2 loss were considered MLH1 deficient. Tumors showing an isolated loss of PMS2 or MSH6 in IHC were considered to present inactivation of the homonymous gene. Confirmative whole section immunohistochemistry (MLH1 and PMS2) was performed on all the cases showing a loss of MLH1 in the TMA and negative methylation analysis.

Methylation-specific multiplex ligation-dependent probe amplification (MS-MLPA) was performed to evaluate the *MLH1* methylation status of the Deng promotor regions C and D. Hypermethylation of either of these regions correlates with expressional silencing of *MLH1* [20,21]. A SALSA MMR MS-MLPA Kit ME011 (MRC-Holland) was used and all MS-MLPA reactions, analyses, and calculations of the methylation dosage ratios were completed according to the manufacturer’s instructions as described before [17]. Tumors with a methylation ratio > 0.15 either in region C or D or both regions were considered hypermethylated [22].

To perform the germline *MLH1* mutational analysis, non-neoplastic tissue blocks (fallopian tube, ovary, gallbladder) were retrieved from the 14 patients with non-methylated MLH1 deficient ECs. DNA was extracted from formalin-fixed paraffin-embedded (FFPE) samples using a GeneRead DNA FFPE Kit (Qiagen, Hilden, Germany) with an optimized protocol for FFPE sample material. Library preparation, sequencing and data analysis were performed as described earlier [23]. The only exceptions for the protocol were that the current in-house panel was designed for *MLH1* gene analysis and the sequencing was performed with IonProton (ThermoFisher Scientific, Waltham, MA, USA). The in-house panel covers all *MLH1* exons and 10 base pairs around the exons. In addition, the panel is designed to detect a Finnish founder mutation *MLH1* del ex16 [24,25]. The average base coverage depth for the analyzed samples was 2332. Subsequently, a SALSA MLPA kit P003 from MRC-Holland (Amsterdam, The Netherlands) was used to screen the DNA samples for other large genomic rearrangements in *MLH1* as described before [26,27]. A dosage quotient of approximately 0.5 was interpreted as a heterozygous deletion of *MLH1* exons. The equivocal cases (dosage quotients of 0.6–0.8) underwent confirmatory testing with the SALSA MLPA kit P248. Variant pathogenicity was assessed using the MMR variant classification criteria by The International Society of Gastrointestinal Hereditary Tumors (InSiGHT) group (www.insight-group.org; accessed on October 25, 2021).

*POLE* exonuclease domain mutation screening of hot spots in exon 9 (c.857C > G, p.P286R; c.890C > T, p.S297F), exon 13 (c.1231G > C, c1231G > T, p.V411L) and exon 14 (c.1366G > C, p.A456P) was performed by direct sequencing as described before [28].

The false negative rate of selective methylation testing was calculated as the proportion of LS patients lying outside the selected age cut-off (≥60 years, ≥65 years or ≥70 years), i.e., patients that would be missed with restrictive screening. Sensitivity was calculated as 1—false negative rate and was based on the assumption that all LS-associated tumors of the *MLH1* mutation carriers presented an MLH1 loss in immunohistochemistry and were nonmethylated. Specificity was calculated as the proportion of non-LS patients (MLH1-negative methylated or nonmethylated tumors in the absence of germline mutation) that based on the patient’s age were correctly classified as probably sporadic. Specificity was only calculated for the clinic-based cohort as the registry-based cohort consisted solely of mutation carriers. Data were analyzed using SPSS version 25 software (IBM Corp., Armonk, NY, USA).

## 3. Results

**Figure 1:**
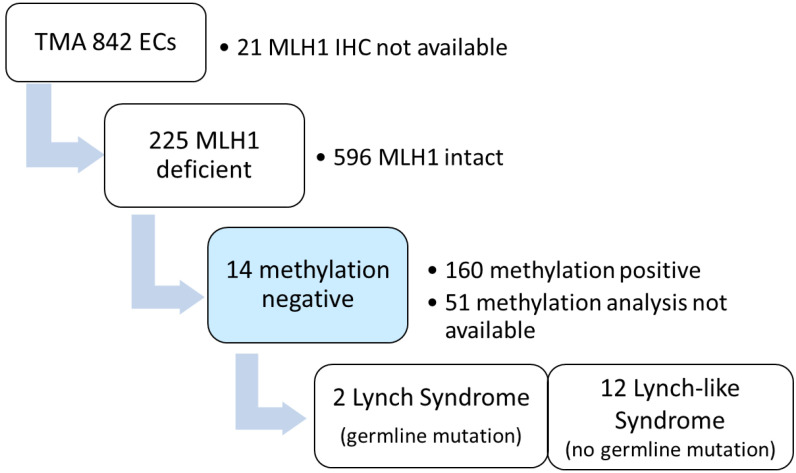
MLH1 immunohistochemistry, methylation analysis and germline mutational analysis performed o 821 unselected EC cases (clinic-based cohort).

MLH1 immunohistochemistry provided conclusive results for 821 cases. A loss of MLH1 (and PMS2) was observed in 225 (27.4%) cases. Seven of these negative cases presented with additional MMR deficiencies (loss of MSH2/MSH6 or MSH6). A *MLH1* promoter methylation test by MS-MLPA was successfully carried out on 174 MLH1-negative tumors. Cases with failed methylation analysis due to a lack of tissue blocks, unsuccessful DNA extraction or low-quality DNA (*n* = 51, 22.7%), were excluded from further analysis (Figure 1).

All cases with multiple MMR anomalies were *MLH1* methylation-linked. Pertinent clinicopathological characteristics of the 174 MLH1-negative cases forming our clinic-based study cohort are shown in Table 1.

Hypermethylation (*MLH1*-Met, methylation ratio > 0.15) was observed in 92% of the MLH1 deficient tumors leaving 14 non-methylated LS suspect cases which were subjected to germline *MLH1* testing. Detailed clinicopathological information on the individual cases included in the germline mutational analysis is shown in Table 2.

NGS-based *MLH1* mutational analysis on non-neoplastic tissue found germline mutation of *MLH1* (LS) in 2/14 (14.3%) of the Lynch-suspect cases. In one case we found exon 16 deletion while the other presented the c.320T > G p.(Ile107Arg) variant (Table 2). These variants were classified as pathogenic. MLPA analysis found no additional cases with germline alterations. The 12/14 patients lacking germline *MLH1* mutations were classified as having Lynch-like syndrome (LLS).

**Figure 2:**
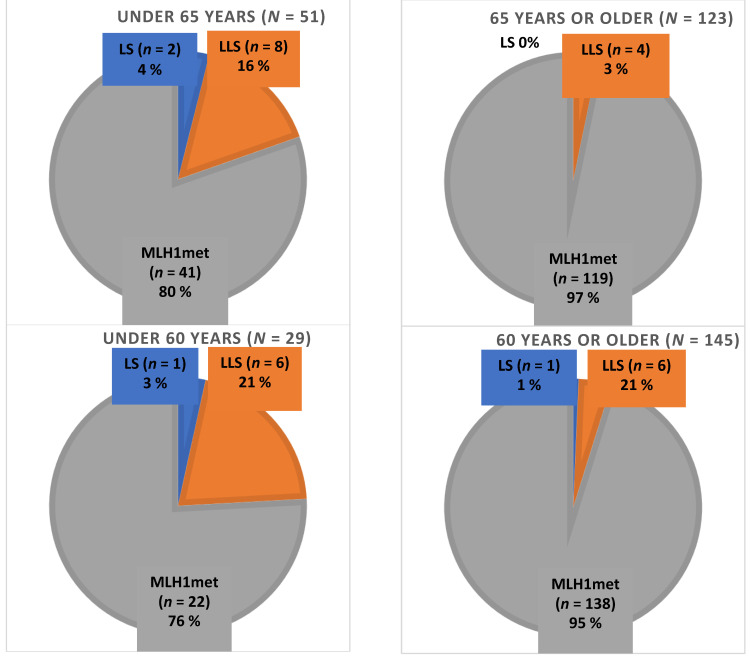
Prevalence of LS, LLS and methylation-linked MLH1-deficient endometrial carcinoma according to age groups (total *n* = 174 patients).

The two confirmed LS patients were diagnosed with EC at the age of 43 and 61 years. The median age at diagnosis of patients with hypermethylation-linked EC and LLS were 72 years and 59.5 years, respectively. The median follow-up time for the clinic-based cohort was 74.0 months (range 2–132). Disease-specific mortality occurred in 37/174 (21.3%) patients in the whole MLH1-deficient tumor cohort including 0/2 (0.0%) of LS cases, 1/12 (8.3%) of LLS cases and 36/160 (22.5%) of the patients with *MLH1* hypermethylation-linked EC. The distribution of LS, LLS and methylation-based MLH1 deficient EC within the various age groups of patients is shown in Figure 2.

**Figure 3:**
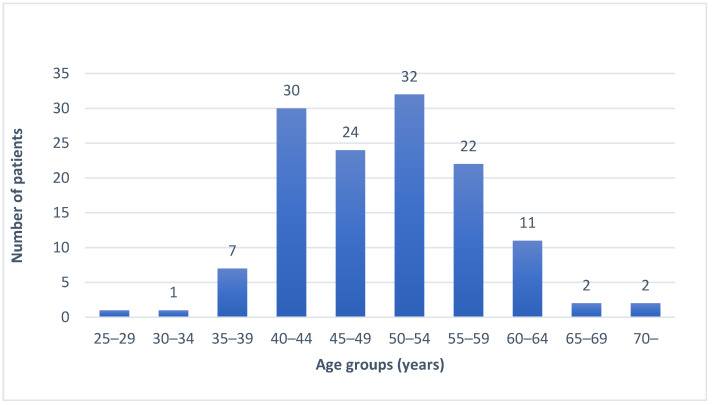
Age distribution of *MLH1* mutation carriers diagnosed with EC in the LS registry-based cohort (*n* = 132).

Age distribution of the patients forming the LS registry-based cohort of *MLH1* mutation carriers is depicted in Figure 3.

In the registry-based cohort 15/132 (11.4%) of the patients were diagnosed with EC at the age of 60 years or older, 4/132 (3.0%) at 65 years or older and 2/132 (1.5%) at 70 years or older. In the age group of >65 years, two of the four ECs were index cases. In the remaining two cases the patients had a previous or concomitant colon carcinoma suggesting a hereditary background of EC. 

As regards the laboratory burden (Table 3), methylation analysis restricted to patients <60 years of age would have excluded 83.3% of these patients from testing compared to universal methylation testing of MLH1-deficient cases. The respective false negative rate for *MLH1*-LS detection was 50% for the clinic-based cohort and 11.4% for the registry-based cohort (sensitivity 50–88.6%). With the cut-off set at 65 years, 70.7% of the patients would be excluded from testing. This methodology would have resulted in a false negative rate for LS detection of 0% and 3% in the clinic-based and registry-based cohorts, respectively (sensitivity 97–100%). With the cut-off set at 70 years, 56.3% of the patients would be excluded from testing, yielding a false negative rate of 0% and 1.5% in the respective cohorts (sensitivity 98.5–100%). The specificity would have been 83.7%, 71.5% and 43.7% with the respective age cut-offs.

The protocol for methylation testing influences the number of referrals to genetic counseling. With universal methylation testing as a triage method, 14/174 (8.0%) of the patients with MLH1 deficient EC (1.8% of all the 770 EC patients with successful immunohistochemistry and methylation analysis) would have been referred to genetic counselling and successive mutational analysis due to suspected *MLH1* mutation carrier status (Table 3). Restricting methylation analysis to patients aged <60, the proportion of patients receiving genetic counselling would have been 7/174 (4%), i.e., a reduction of 50% compared to the triage by universal methylation testing. The corresponding proportion of referrals would be 10/174 (5.7%) for the cut-off <65 years (reduction of 28.6%) and 13/174 (7.4%) for <70 years (reduction of 7.1%) (Table 3). 

## 4. Discussion

The goal of LS screening in EC patients is to provide future cancer surveillance for the newly diagnosed patient as well as germline variant testing for her at-risk relatives. LS screening protocols based on clinical criteria (Amsterdam I and II, Bethesda) suffer from limited sensitivity as they fail to identify over 40% of LS carriers [29,30,31]. The most accurate and cost-effective method of screening appears to be tumor-based MMR immunohistochemistry and *MLH1* methylation analysis, which identify patients that presumably benefit from germline mutational testing [8,32,33,34]. Given that MMR deficiency is common in EC and, LS is likely to be rare in elderly patients, it is tempting to propose a selective methylation analysis based on patient age as a tool to improve the cost-effectiveness of LS screening. 

In our clinic-based cohort of 174 patients with MLH1-negative EC, 2 of the 14 non-methylation linked cases (14.3%) were associated with pathogenic germline mutations corresponding to LS. In a previous meta-analysis, the corresponding finding was 22.4% [30]. Our lower relative frequency of LS-related EC may partly be explained by active gynecological surveillance and prophylactic surgery that has been offered to LS carriers in Finland after 1995 [35]. The remaining cases lacking *MLH1* methylation and germline mutations are generally classified as having Lynch-like syndrome, which may be caused by double somatic mutations [36,37]. Family members of patients with LLS appear to have an increased overall cancer risk, although the risk is lower than in families with LS [38]. As regards the clinical characteristics, such as age and BMI, EC patients with LLS appear to be more similar to patients with MMR proficient EC than to those with LS [39]. Given the variable risk of inheritance and uncertain clinical significance of LLS, it is not clear whether these patients should be dealt with similarly to LS patients or sporadic MMRd cases [38,39].

To determine the optimal age cut-offs for targeted testing, data on the age-specific incidence of EC in LS patients is needed. Given the MMR gene-specific (*MLH1*, *PMS2*, *MSH2*, *MSH6*) differences regarding the age of cancer occurrence [3], it is essential to determine optimal screening protocols separately for each MMR protein abnormality detected by immunohistochemistry. In a previous registry-based LS study by Møller et al., all of the 110 EC cases occurring in female carriers of the *MLH1* mutation were diagnosed before the age of 60 years [3]. By contrast, in *MSH2* and *MSH6* mutation carriers, EC cases were found up until the end of the observation period (70 years of age) [3].

It should be noted that registry-based cancer syndrome studies may theoretically create bias due to selective inclusion, as most of the patients have been referred to genetic testing based on clinical criteria. This may result in an overrepresentation of families carrying a stronger cancer risk with possibly an earlier age of occurrence; however, this appears not to be the case as regards *MLH1* mutation carriers as in the population-based Australian National Endometrial Cancer Study, no *MLH1*-LS cases were found in EC patients above 60 years of age [14]. Similarly, in our clinic-based cohort of 174 patients, only one MLH1-LS patient was diagnosed with EC at the age of 60 or older (61 years). In the Finnish Lynch registry-based cohort, 11.4% of ECs diagnosed in *MLH1* mutation carriers occurred in patients aged 60 years or older, 3.0% in 65 years or older and 1.5% in patients 70 years or older. The prevalence of various LS-related founder mutations varies according to geographical areas, which may explain the small observed differences in the age-specific prevalence of EC arising in *MLH1* mutations carriers in different countries [40]. Two of the Finnish LS patients with an age >65 years would have been referred to genetic counselling based on the revised Bethesda criteria regarding the presence of other metachronous or synchronous LS tumors. Importantly, the current guidelines recommend genetic counseling for all patients with familial history highly suspicious of Lynch syndrome (independent of the MMR status), which further improves the sensitivity of selective tumor-based screening strategies [7,16].

The main goal of our study was to explore the trade-off between a reduced testing effort and the sensitivity of targeted LS screening methods. We compared the universal testing of EC patients with selective testing using various age cut-offs. The findings were confirmed in a large registry-based cohort. Based on our results, restricting methylation analysis to patients under 65 years of age would considerably reduce the number of tests performed, excluding 70.7% of the patients with MLH1-negative EC from testing. The corresponding false negative rate, i.e., the proportion of missed MLH1-LS patients, would have been 0–3%. Methylation analysis restricted to patients <60 years would have excluded 83.3% of these patients from testing. A respective false negative rate for MLH1-LS detection was 11.4% for the registry-based cohort. Combining molecular analysis and clinical criteria would further increase the sensitivity of targeted LS screening in EC patients.

A major limitation of our study was the low number of LS patients in the clinic-based cohort, which was due to its unselected nature and the expectedly low prevalence of *MLH1*-LS; however, our results were confirmed in a sizable registry-based cohort. A low prevalence of germline mutations further supports the usefulness of selective methylation testing. The goal of our study was to investigate the possibility of age-selective methylation testing. Thus, we excluded methylation-unrelated cases presenting an isolated loss of PMS2 or MSH2/MSH6 deficiency. As a consequence, our estimates regarding immunohistochemistry and methylation-based Lynch syndrome screening only apply to patients with MLH1-negative EC. As regards the MMR protein deficiencies other than of MLH1, the prevalence of MSH2/MSH6/PMS2 losses in EC are significantly lower than that of MLH1 loss (1–3% vs. 20%, respectively) [1]. Considering also the older age of the MSH2/MSH6 mutation carriers diagnosed with EC, age-selective screening is not necessary or feasible in the case of MMR deficiency due to other than MLH1 protein loss [3].

To overcome the limitations related to the TMA-based MMR immunohistochemistry, an experienced pathologist carefully selected the tumor areas for punching in order to exclude precursor lesions and poorly fixed areas, which may have produced false negative and positive results, respectively. Further, we included four punch biopsies from each tumor to increase the representability of our TMA results. In addition, all the non-methylated MLH1 negative cases were confirmed by whole section immunohistochemistry. Mutational screening may pose the dilemma of how to address variants of unknown/uncertain significance (VUS). We adopted the MMR variant interpretation criteria established by the InSiGHT group. Germline data were available for the non-methylated cases only, but our estimates on the LS prevalence can be considered accurate given that false negative immunohistochemical results (due to functional mutations not compromising antigen expression) are rare in MMR tumors and *MLH1* methylation is extremely rare in LS associated ECs [41]. The age-specific prevalence of EC arising in *MLH1* mutation carriers may present geographical variation according to the prevalence of specific *MLH1* mutations.

## 5. Conclusions

Based on our findings, age-guided targeted *MLH1* methylation analysis could improve the cost-effectiveness of LS screening procedures. In our Finnish cohort, the age cut-off set at 65 years produced a significant reduction in testing effort and an acceptable false negative rate. Validation of our findings is needed in other populations of EC patients.

## Figures and Tables

**Table 1:** Clinicopathologic data *(n* = 174 *).

Age (Years) (Median, Range)	71.5 (43–94)
Histology (number of cases, percent)	
Endometrioid carcinoma	160 (92.0)
Clear cell carcinoma	4 (2.3)
Serous carcinoma	1 (0.6)
Undifferentiated carcinoma	6 (3.4)
Carcinosarcoma	3 (1.7)
Grade (number of cases, percent) (For endometrioid only, *n* = 160)	
Grade 1 or 2	122 (76.3)
Grade 3	38 (23.8)
FIGO 2009 stage (number of cases, percent)	
IA	82 (47.1)
IB	39 (22.4)
II	15 (8.6)
IIIA	13 (7.5)
IIIB	1 (0.6)
IIIC1	17 (9.8)
IIIC2	6 (3.4)
IVA	0 (0.0)
IVB	1 (0.6)
Adjuvant therapy (number of cases, percent)	
No adjuvant therapy	19 (10.9)
Vaginal brachytherapy	76 (43.7)
Whole pelvic radiotherapy	31 (17.8)
Chemotherapy	17 (9.7)
Chemotherapy and whole pelvic radiotherapy	31 (17.8)

* Cases with MLH1-deficient tumor and successful methylation analysis.

**Table 2:** Clinicopathological characteristics of patients with non-methylated MLH1-deficient endometrial carcinoma.

*MLH1* Germline Mutation (NM_000249.4)	Age (Years)	Histology	FIGO 2009 Stage	*POLE* Mut
c.320T > G, (p.Ile107Arg)	43	Endometrioid G3	IA	
exon 16 deletion	61	Clear cell carcinoma	IA	wt
no	48	Clear cell carcinoma	IA	wt
no	51	Endometrioid G1–2	IA	wt
no	56	Endometrioid G1–2	IA	wt
no	57	Endometrioid G3	IA	
no	59	Endometrioid G3	IIIC1	wt
no	59	Endometrioid G3	IA	wt
no	60	Endometrioid G3	IA	wt
no	61	Endometrioid G1–2	IA	wt
no	66	Clear cell carcinoma	IIIa	mut
no	67	Endometrioid G1–2	IA	wt
no	69	Endometrioid G1–2	IB	
no	77	Endometrioid G1–2	IA	wt

**Table 3:** Comparison of universal and selective *MLH1* methylation testing methods according to different age cut-offs.

Outcome	Universal Met Testing (*n*, %)	Cut-Off <70 Years (*n*, %)	Cut-Off <65 Years (*n*, %)	Cut-Off <60 Years (*n*, %)
Patients excluded from methylation testing *	0/174 (0.0)	98/174 (56.3)	123/174 (70.7)	145/174 (83.3)
Patients excluded from genetic testing *	0/14 (0.0)	1/14 (7.1)	4/14 (28.6)	7/14 (50)
LS/Met-tested cases (*n*, %) *	2/174 (1.1)	2/76 (2.6)	2/51(3.9)	1/29 (3.4)
False negative rate (LS) *	0/2 (0.0)	0/2 (0.0)	0/2 (0.0)	1/2 (50)
False negative rate (LS) **	0/132 (0.0)	2/132 (1.5)	4/132 (3.0)	15/132 (11.4)

LS = Lynch syndrome, Met-tested = methylation tested, * clinic-based cohort, ** registry-based cohort.

## Data Availability

All relevant data are within the paper.
